# Citrate Pharmacokinetics in Critically Ill Patients with Acute Kidney Injury

**DOI:** 10.1371/journal.pone.0065992

**Published:** 2013-06-18

**Authors:** Yin Zheng, Zhongye Xu, Qiuyu Zhu, Junfeng Liu, Jing Qian, Huaizhou You, Yong Gu, Chuanming Hao, Zheng Jiao, Feng Ding

**Affiliations:** 1 Division of Nephrology, Huashan Hospital, Fudan University, Shanghai, China; 2 Clinical Pharmacy Laboratory, Huashan Hospital, Fudan University, Shanghai, China; University of São Paulo School of Medicine, Brazil

## Abstract

**Introduction:**

Regional citrate anticoagulation (RCA) is gaining popularity in continous renal replacement therapy (CRRT) for critically ill patients. The risk of citrate toxicity is a primary concern during the prolonged process. The aim of this study was to assess the pharmacokinetics of citrate in critically ill patients with AKI, and used the kinetic parameters to predict the risk of citrate accumulation in this population group undergoing continuous veno-venous hemofiltration (CVVH) with RCA.

**Methods:**

Critically ill patients with AKI (n = 12) and healthy volunteers (n = 12) were investigated during infusing comparative dosage of citrate. Serial blood samples were taken before, during 120 min and up to 120 min after infusion. Citrate pharmacokinetics were calculated and compared between groups. Then the estimated kinetic parameters were applied to the citrate kinetic equation for validation in other ten patients’ CVVH sessions with citrate anticoagulation.

**Results:**

Total body clearance of citrate was similar in critically ill patients with AKI and healthy volunteers (648.04±347.00 L/min versus 686.64±353.60 L/min; P = 0.624). Basal and peak citrate concentrations were similar in both groups (*p* = 0.423 and 0.247, respectively). The predicted citrate curve showed excellent fit to the measurements.

**Conclusions:**

Citrate clearance is not impaired in critically ill patients with AKI in the absence of severe liver dysfunction. Citrate pharmacokinetic data can provide a basis for the clinical use of predicting the risk of citrate accumulation.

**Trial Registration:**

ClinicalTrials.gov Identifier NCT00948558

## Introduction

Regional citrate anticoagulation (RCA), which restricts its powerful antithrombotic effect to the extracorporeal circuit, has been increasingly adopted as an alternative to heparin in critically ill patients with high risks of bleeding. Besides a significant decrease in bleeding [1]–[2], RCA improves the patency [3], biocompatibility [4] and extends the life-span of dialyzers [5]–[6]. Moreover, an additional important point deserves attention: during hemodialysis using citrate anticoagulation, leukocyte activation and calcium-mediated activation of complements are suppressed [7]. A large randomized controlled trial has shown that this mode of anticoagulation, compared to nadroparin, resulted in lower mortality of critically ill patients on CRRT, particularly benefiting the patients who suffered from sepsis or severe multiple organ failure, after surgery, beyond the effect of less bleeding [8]. However, citrate accumulation, characterized by life-threatening hypocalcemia and metabolic acidosis, is a major safety concern which inhibits its broad clinical acceptance.

Under physiologic circumstances, citrate is metabolized in the liver, skeletal muscle and renal cortex [9]. The study of Kramer et al [10] confirmed the role of hepatic handling of citrate by demonstrating approximately 50% reduced citrate clearance in cirrhotic patients. In the case of kidney injury, both the absence of metabolically active tissue mass and the uremic state would contribute to impairment of citrate metabolism. Unexpectedly, Bauer et al [11] reported the citrate clearance was not impaired in patients with chronic renal failure. It is unknown whether critically ill patients with acute kidney injury (AKI) have difficulty with citrate metabolism.

During continuous dialysis modalities, a larger total load of citrate is delivered over a prolonged treatment process compared to intermittent hemodialysis. The early and available assessment of citrate accumulation during RCA-CRRT is of high relevance. However, plasma citrate monitoring during CRRT is not routinely available. Though several indirect markers of citrate accumulation such as pH, anion gap, total calcium, ionized calcium or total/ionized calcium ratio have been suggested [8], [10], [12]–[13], all these parameters correlate poorly with measured citrate levels except the total/ionized calcium ratio, which unfortunately could not identify all the cases of citrate accumulation [10], [13]–[14].

To improve safety and expand the use of RCA, in this study, we first investigated the citrate pharmacokinetics (PK) in critically ill patients with AKI. Then, we implemented the pharmacokinetic parameters to prospectively evaluate the citrate level in this patient group during the continuous veno-venous hemofiltration (CVVH).

## Materials and Methods

### Subjects

Critically ill patients with AKI, aged 18–75 yrs, requiring CRRT, were included in this study. Exclusion criteria were severe liver injury or liver failure, marked alkalosis (PH >7.5), blood or plasma transfusion within 24 hours prior to the study, use of citrate-containing medications, dying within 48 hours. A total of 22 critically ill patients with AKI were included. Twelve of 22 patients were involved for the investigation of the citrate pharmacokinetics, and as a comparative group, twelve age-and gender matched healthy volunteers without regular medications and a normal renal function according to the Modification of Diet in Renal Disease (MDRD) formula were enrolled. The healthy volunteers were recruited from the local community and Fudan University. Every healthy volunteer who took part in the study participated of their own will. They would have the following benefits: receiving a free physical examination and laboratory tests for blood cell counts, routine blood biochemical values, receiving financial compensation and providing health counseling. For the application of citrate pharmacokinetics, the CVVH treatment sessions of the other 10 critically ill patients were investigated. This clinical study was designed, implemented and reported in accordance with the Declaration of Helsinki and local regulations. The study protocol was approved by the Ethics Committee of Huashan Hospital, Fudan University (approval No. KY2009-98) and written informed consent was obtained from participants themselves or their close relatives before enrolment. We confirm that all potential participants who declined to participate or otherwise did not participate were eligible for treatment (if applicable) and were not disadvantaged in any other way by not participating in the study.

### Citrate Anticoagulation in Patients

All the patients were treated with CVVH using a standard citrate anticoagulation, which the trisodium citrate solution (4%, 136 mmol/L) was infused into the arterial line prior to the blood pump at a dose of 3.7 mmol/L of plasma flow, and the calcium chloride solution (5%, 340 mmol/L) was supplemented into the venous return. For citrate pharmacokinetic investigation, citrate was delivered for two hours and then stopped infusing in the following two hours while a low dose of heparin was given for anticoagulation instead. Thereafter, RCA continued. For citrate PK application, the other ten critically ill patients underwent citrate anticoagulation during the whole process of CVVH.

### Citrate Infusion and Calcium Substitution in Healthy Volunteers

All the healthy volunteers were given intravenous 4% trisodium citrate solution at the infusion rate of 30 mmol/h for 2 hours. Meanwhile, the diluted calcium chloride solution was replaced via a separate peripheral vein to maintain the ionized calcium concentration greater than 0.9 mmol/L.

### CRRT Protocol

CVVH was performed in pre-dilution mode using the Diapact CRRT machine (B. Braun, Germany) set with a HIPS 15 hemofilter (effective surface area 1.5 m^2^, B. Braun, Germany). The blood flow rate was set at 150–200 mL/min. The substitution fluid was delivered at the rate of 4 L/h, which contained 121 mmol/L Na^+^, 3.2 mmol/L K^+^, 0.75 mmol/L Mg^2+^, 250 mg/dL dextrose, and 18 mmol/L bicarbonate. With heparin anticoagulation, the composition of substitution fluid contained 142 mmol/L Na^+^, 3.2 mmol/L K^+^, 1.5 mmol/L Ca^2+^, 0.75 mmol/L Mg^2+^, 250 mg/dL dextrose, and 34 mmol/L bicarbonate. The ultrafiltration flow rate was determined according to the patients’ condition. The blood flow rate, the ultrafiltration rate and the replacement fluid rate were recorded at each blood sampling point.

### Laboratory Tests

To evaluate the citrate PK, blood samples were collected from the arterial line prior to the citrate infusion for the systemic citrate concentration testing at baseline (predose) and after 10, 20, 40, 60, 120 (end of infusion), 125, 130, 140, 160, 180, 210 and 240 min postdose. In addition, 10 min after the end of citrate infusion (120 min), blood samples were taken simultaneously from both pre-filter and post-filter for the measurement of citrate clearance by filter.

In the process of the other 10 patients’ CVVH treatment sessions with citrate anticoagulation, blood samples were obtained for the measurement of systemic citrate concentration at 0 h, 1 h, 2 h, 3 h, 6 h, 8 h.

Baseline full blood counts were measured by a Sysmex XE-2100 hematology analyzer (Sysmex, Kobe, Japan), and baseline blood chemical values were measured by a Hitachi 7600-020a autoanalyzer (Hitachi, Tokyo, Japan). Ionized calcium was checked immediately by the i-STAT 300 analyzer every two hours of the treatment (Abbott Laboratories, Illinois, USA).

The level of citrate in plasma was determined using a high-performance liquid chromatography (HPLC) method with ultraviolet detection at 210 nm (Agilent 1100 series, USA). Plasma specimen (100 µm) were deproteinized by centrifugal ultrafiltration at 6000 g for 45 min using Sartorius Vivaspin 20 (3 Kda MWCO polyethersulfone membrane). The obtained ultrafiltrate was stored at −20°C until use. The HPLC system consisted of a Model G1311A quaternary pump, a Model G1322A degasser, a Model G1313A autosampler and a Model G1314A variable wavelength detector. The separation for citrate was performed on a Kromasil 100-3.5 C18 column (5 µm, 2.1×150 mm i.d.) with a guard column(10 µm, 20×2.1 mm i.d.) by 10 mM potassium phosphate buffer as mobile phase (pH 2.4) at a flow rate of 0.15 ml/min. The method was found to be linear in the range of 10.9–5000 µmol/L (r = 0.996), with a detection limit (signal/noise ratio of 3) of 3.2 µmol/L. The mean recoveries were 96.2% for three spiked level. Both inter-assay and intra-assay coefficients of variation were less than 6% and 11%, respectively.

### Pharmacokinetic Calculations

Pharmacokinetic analysis was performed with the software package WinNonlin Program (Version 4.1, Pharsight Corporation, Mountain View, USA) using non-compartmental approaches. Area under the concentration-time curve (AUC) was calculated from non-fitted data by employing the linear trapezoidal rule. The time to peak (Tmax) and the peak concentration (Cmax) were measured directly from the concentration-time curve. Volume of drug distribution (Vd) and total drug clearance (Cl) were calculated by use of standard formulae:
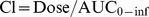


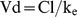
where k_e_ is the elimination rate constant. Because of citrate exclusion by red blood cells [15], the plasma water clearance of citrate by filter (Cl_fiter_) was calculated by the formula:



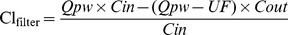



UF is the ultrafiltration flow rate, and Cin and Cout are plasma water concentrations of citrate at the inlet and the outlet of the filter, respectively. Qpw can be calculated by the following equation: 
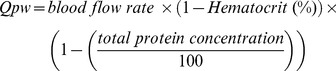
 Thereby, the total body citrate clearance of a patient (Cl_body_) was obtained by subtracting the citrate extracorporeal clearance from the total citrate clearance.

### Calculations of Systemic Citrate Concentrations

After obtaining the citrate pharmacokinetic parameters, we applied them to the citrate kinetic modeling equation [16] to predict the systemic citrate level in the other ten critically ill AKI patients undergoing RCA-CVVH. This kinetic model integrates all the citrate fluxes parameters in the patients and the blood circuit, on the basis of citrate exclusion by red blood cells, rapid hemofilter citrate clearance and citrate infusion at a fixed ratio to arterial plasma flow. The systemic citrate level can be described as a function of the RCA prescription and the rate of body metabolism of citrate:
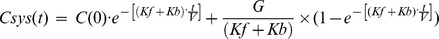
where Csys(t) (mmol/L) is the citrate level in systemic plasma, C(0) (mmol/L) is plasma citrate at t = 0, V (L) is volume of citrate distribution, Kf (L/h) is plasma clearance of citrate on the filter, Kb (L/h) is plasma clearance of citrate in the body, and G (mmol/h) is citrate load.

According to the pharmacokinetic principle of CRRT [17], the citrate removal by filter in pre-dilution hemofiltration modality can be estimated by the following formula:
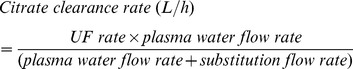



### Data Analysis

The required sample size for citrate PK study was estimated by the equation: n_1_ = n_2_ = 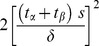 [18], where α = 0.05, β = 0.20 and the values of s and δ were assumed based on the data of the published papers [10]–[11]. Pharmacokinetic parameters are expressed as geometric mean ± SD. Other data are presented as mean ± SD or median with inter-quartile range. Normal distribution of samples was tested with the Kolmogorov-Smirnov test. Comparison between groups was performed using independent t-test or Mann Whitney U test for continuous variables according to data distribution, and Pearson chi test or Fisher exact test for categorical variables. Differences between predicted and measured values were calculated as predicted – measured and tested for significant deviation from zero by means of a two-tailed one-sample t test. Bland-Altman plots were used for analyzing the agreement of predicted and measured values and the systematic bias. The differences were considered statistically significant with p value <0.05 (two tail tests). Statistical analysis was performed using a statistical software package (SPSS), version 16.0.

## Results

### Baseline Characteristics

The clinical characteristics of patients involved for citrate PK study are summarized in Table 1. Healthy volunteer group did not differ from the patient group with respect to age, gender proportion and body mass index. The demography and baseline laboratory parameters of both groups are provided in Table 2. For implementation of the citrate pharmacokinetic parameters, the other ten critically ill AKI patients with mean age (± SD) 57.3 (±13.8) years old and mean Sepsis-related Organ Failure Assessment (SOFA) scores [19] (± SD) 11.0 (±3.6) were enrolled.

**Table 1:** Clinical characteristics of patients involved in the citrate pharmacokinetics study.

Patient	Age/Sex	Causes of Admission	RIFLE Class	Vasopressor (Y/N)			Mechanical Ventilation (Y/N)	SOFA Scores	
1	31/M	Severe acute pancreatitis	Fc		N	Y		8	
2	70/M	Heart failure	Fc		N	N		4	
3	50/M	Rhabdomyolysis	Fc		N	N		8	
4	50/M	Cerebral trauma	Fc		Y	Y		16	
5	73/M	Acute obstructive suppurative cholangitis	Fc		Y	Y		12	
6	43/M	Cerebral hemorrhage	Fc		Y	Y		12	
7	37/M	Cerebral trauma	Ic		Y	Y		14	
8	45/F	Cerebral hemorrhage	Fc		N	N		7	
9	72/M	Cerebral infarction	Fc		N	Y		12	
10	71/M	Cerebral trauma	Ic		Y	Y		19	
11	64/F	Liver transplantation, after surgery	Ic		Y	Y		9	
12	18/F	Polymyositis, Rhabdomyolysis	Ic		N	Y		8	

**Table 2:** The demographic and baseline laboratory parameters of patient group and healthy volunteer group.

		Normal Range	Critically Ill Patients with AKI (n = 12)	Healthy Volunteers (n = 12)	*p* value
Age(y)		/	52.7±16.9	49.2±4.2	0.51
Gender(male/female)		/	9/3	8/2	1.0
Body Mass Index (kg/m^2^)		18.5–23.9	23.5±2.9	22.6±2.3	0.46
White blood cell (×10^9^/L)		4.5–11.0	9.9±4.3	5.7±1.1	0.006
Hematocrit (%)		36–53	28.0±8.1	41.9±3.5	<0.001
Hemoglobin (g/L)		120–160	93.3±28.9	146.4±13.9	<0.001
Platelet (×10^9^/L)		100–300	142.1±116.0	180.4±45.9	0.36
ALT (U/L)		<50	26 (10.5–120.3)	20.0 (15.0–43.5)	0.60
AST (U/L)		<30	41.5 (28.5–164.5)	22.0 (19.0–27.0)	0.009
Bilirubin (mmol/L)		3.4–20.4	20.5±29.7	14.0±6.4	0.51
Albumin (g/L)		35–50	31 (27–35)	43.0 (42–45)	<0.001
Glucose (mmol/L)		3.9–5.8	7.7 (6.4–10.3)	5.1 (4.9–5.2)	0.001
Ca^2+^ (mmol/L)		2.1–2.6	2.1 (1.9–2.3)	2.3 (2.2–2.4)	0.42
HCO_3_^−^(mmol/L)		22.0–28.0	20.9 (18.4–23.6)	27.0 (26.1–27.9)	0.001
BUN (mmol/L)	2.5–7.0		22.9 (21.2–30.4)	5.2 (5.0–6.3)	<0.001
Creatinine (µmol/L)		50–130	332 (179–484)	72 (66–80)	<0.001

ALT: alanine aminotransferase; AST: aspartate aminotransferase; BUN: blood urea nitrogen.

Data are represented as mean ± SD or median with interquartile range.

### Citrate Pharmacokinetics

**Figure 1:**
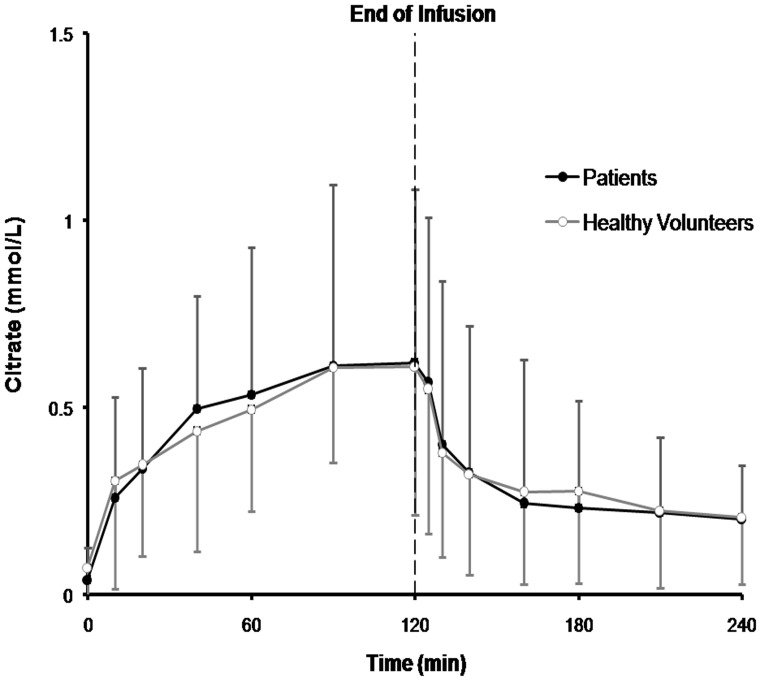
Plasma citrate concentrations (mean ± SD) during 120 min citrate infusion and 120 min after the end of citrate infusion in critically ill patients with AKI (n = 12) and in healthy volunteers (n = 12).

Main pharmacokinetic parameters are displayed in Table 3. Total citrate load between two groups were comparative (63.7±9.1 mmol in patients group vs. 57.1±10.5 mmol in healthy group, P = 0.13). Basal citrate level was not increased in critically ill patients with AKI (p = 0.42). The mean plasma citrate concentration-time curves of both groups during 120 min citrate infusion and 120 min after the end of citrate infusion are shown in Figure 1. No significant difference was observed in AUC, citrate clearance and peak plasma citrate concentration. The mean citrate plasma water clearance by filter was 44.6±7.3 ml/min, accounting for 11% of the total citrate clearance.

**Table 3:** Citrate pharmacokinetics.

	Critically Ill Patients with AKI (n = 12)	Healthy Patients (n = 12)	*p* value
AUC_0-t_ (mmol·min/L)	79.7±95.5	69.9±66.6	0.60
AUC_0-inf_ (mmol·min/L )	86.8±113.8	87.5±95.5	0.95
t_max_ (min)	110.0±18.1	106.6±21.7	0.78
Vd (L)	21.0±19.5	50.6±21.7	0.09
Cl_body_(ml/min)	648.0±347.0	686.6±353.6	0.62
C_baseline_ (mmol/L)	0.01±0.13	0.02±0.04	0.42
C_max_ (mmol/L)	0.62±0.46	0.56±0.45	0.25
Total dose (mmol)	63.7±9.1	57.1±10.5	0.13

AUC0-t: area under the concentration time-curve from time 0 to t hour, where t was the last time point of the interval with a measurable drug concentration; AUC0-inf: area under the concentration time-curve from time 0 to infinity; tmax: time to maximum concentration; Vd: volume of distribution; Clbody: body clearance; Cbaseline: baseline concentration; Cmax: maximum concentration.

Data are represented as geometric mean ± SD.

### Measured and Predicted Systemic Citrate Concentrations

**Figure 2:**
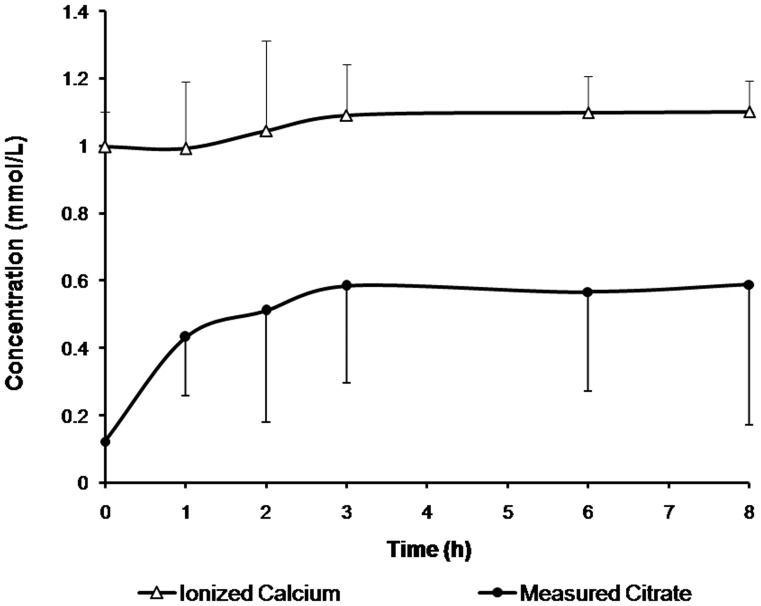
Measured citrate and ionized calcium curves during the first 8 hours of CVVH using citrate anticoagulation while the blood rate were set at 150–200 ml/min and the substitution fluid were at the rate of 4 L/h (n = 10 treatments).

**Figure 3:**
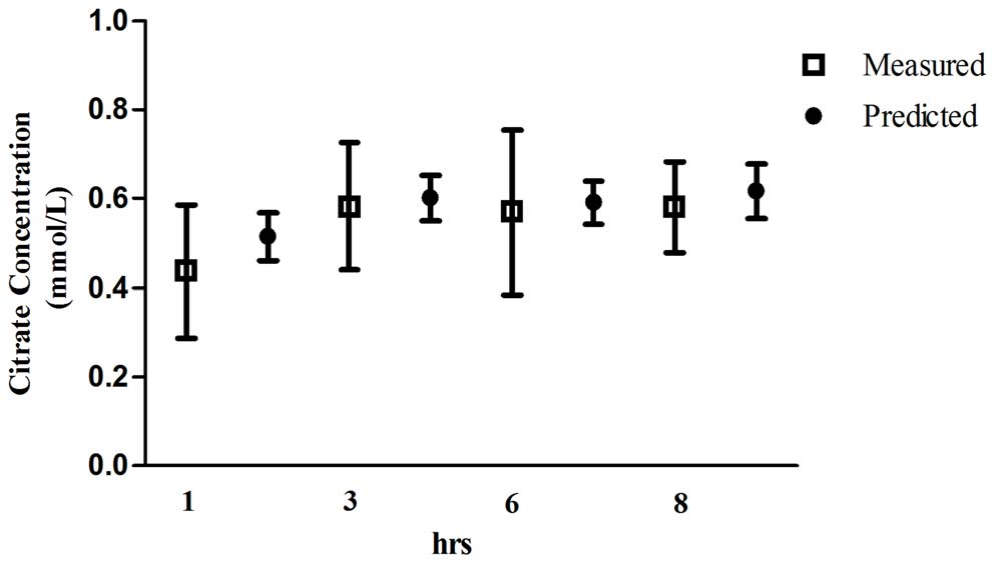
The mean measured and predicted systemic citrate concentrations with 95% confidence intervals at 1 h, 3 h, 6 h and 8 h (vertical lines).

**Figure 4:**
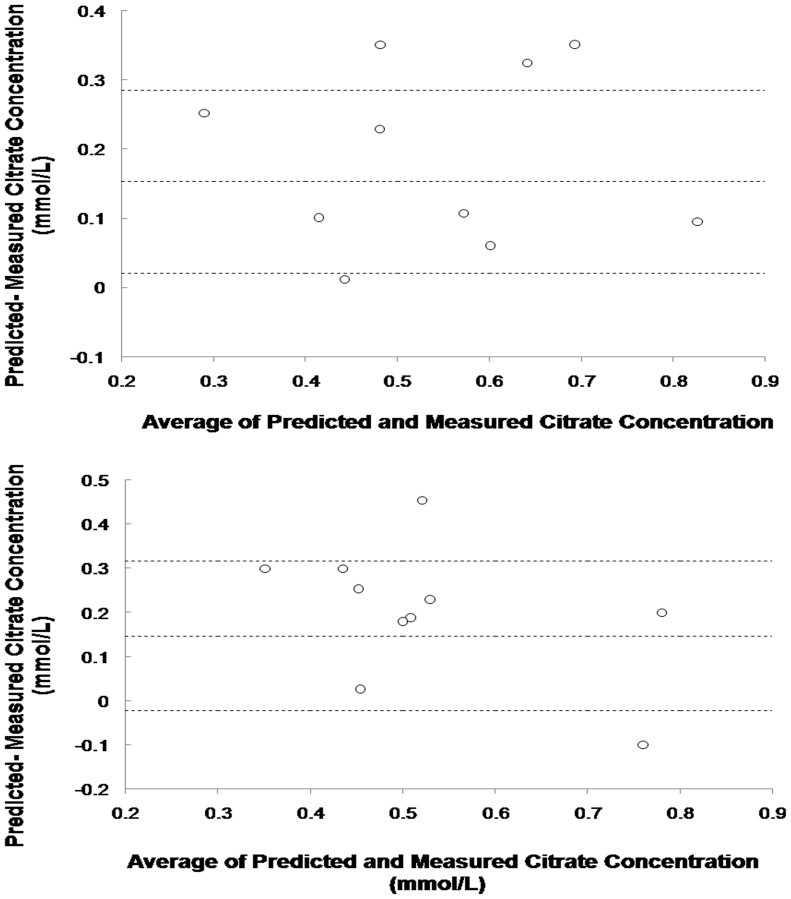
Comparison of predicted and measured systemic citrate concentrations at the 3rd hour (upper) and 8th hour (lower) of CVVH with RCA. The dotted lines represented the 95% confidence intervals of the differences of predicted and measured systemic citrate concentrations.

Measured systemic citrate and ionized calcium concentrations are shown in Figure 2. All the measured citrate concentrations were below 1 mmol/L and systemic ionized calcium levels were maintain at the normal range. The mean measured and predicted systemic citrate concentration with 95% confidence intervals of concentrations are represented in Figure 3. Measured and predicted systemic citrate concentrations at the third hour were 0.58±0.29 mmol/L and 0.60±0.08 mmol/L, respectively (difference -0.15±0.06, 95% confidence interval, CI, −0.28 to −0.08). At the 8th hour of treatment, the measured and predicted systemic citrate concentration was 0.58±0.42 mmol/L and 0.61±0.07 mmol/L, respectively (difference −0.15±0.08, 95% confidence interval, CI, −0.02 to 0.32). Bland-Altman plots revealed no systematic bias in these predictions (Figure 4).

## Discussion

Citrate is mainly metabolized in two organs, the liver and the kidneys [9]. It is tempting to say that liver or kidney dysfunction may be associated with citrate metabolism impairment. Studies have shown that citrate metabolism is impaired in patients with acute liver failure [20] and critically ill cirrhotic patients [10]. Bauer et al [11] investigated that citrate clearance is not compromised in patients with chronic renal failure. Despite the increasing application of CRRT with RCA in critically ill AKI patients, no systematic study of citrate kinetics has been performed in this patient group.

Our data demonstrate that citrate clearance is not impaired in critically ill patients with AKI in absence of severe liver dysfunction, compared to the healthy volunteers. Citrate dose, basal and peak citrate concentrations, AUC were not different between groups. It was suggested that the citrate can be successfully metabolized in critically ill patients with AKI. It could be explained by hepatic predominant metabolism of citrate. Of note, although the distribution volumes of citrate were not statistical different between two groups, there was a lower tendency in patients group. This parameter in our patient group was similar to the result reported by Kramer et al, in contrast to the result of Kozik-Jaromin’s study [21] which concluded that citrate was distributed in a larger volume (40L). This may contributed to the time of citrate infusion (120 min in our study and Kramer study compared to 240 min in Kozik-Jaromin’s study), since a disposition of a substance is a time-dependent process. Furthermore, the different clinical characteristics of the studied population may impact the distribution patterns, especially in the condition of unstable hemodynamic state, varying degrees of edema or dehydration.

In clinical setting, the routine test of plasma citrate concentration is not available. Instead, the reliability of several indirect markers of citrate accumulation, such as: the arterial pH, anion gap, or total calcium-ionized calcium ratio, are controversial and repeated monitoring increases the complexity of RCA procedure. What’ more, up to date there has no available and convenient way to assess the risk of citrate accumulation before treatment at patient’s bed. In the current study, we validated the citrate kinetic equation proposed by Yee et al in the critically ill patients with AKI during RCA-CVVH. Because none of the patients developed citrate accumulation, the utility of the model to predict citrate toxicity could not be reflected by the protocol applied. Nevertheless, our data demonstrated that the citrate kinetic model based on the citrate PK is a useful tool to predict the development of citrate concentrations in the procedure of RCA-CVVH. Given the CRRT treatment parameters and citrate PK, prior to the treatment, we could tell whether the patient would have high citratemia and whether the patient’s RCA-CVVH protocol would be safe and sound. For instance, to the same patients, when the blood flow is set at 200 ml/min and the substitution fluid is at the rate of 2L/h, the predicted maximum systemic citrate concentrations will exceed 1.0 mmol/L, which is not quite safe. In this case, we should optimize the RCA-CVVH protocol to improve safety, e.g: increasing the effluent fluid rate to increase the citrate clearance in the extracorporeal circuit.

The current study suffers several limitations. First, for ethic concern and sample size limitation, in this study, critically ill patients with various RIFLE stages were not stratified. Second, the fitness of citrate kinetic equation was just tested in the mode of pre-dilution CVVH. It awaits further clinical validations in other modalities of CRRT.

### Conclusions

Citrate clearance is not impaired in critically ill AKI patients in the absence of severe liver dysfunction. Citrate pharmacokinetic data provides a basis for the clinical use of citrate kinetic equation to estimate the risk of citrate accumulation and design safety RCA protocols.
